# Eight Priorities for Improving Primary Care Access Management in Healthcare Organizations: Results of a Modified Delphi Stakeholder Panel

**DOI:** 10.1007/s11606-019-05541-2

**Published:** 2019-11-14

**Authors:** Lisa Rubenstein, Susanne Hempel, Margie Danz, Danielle Rose, Susan Stockdale, Idamay Curtis, Susan Kirsh

**Affiliations:** 1 Evidence-Based Practice Center, RAND Corporation, Santa Monica, CA USA; 2 University of California Los Angeles David Geffen School of Medicine, Los Angeles, CA USA; 3 University of California Los Angeles Fielding School of Public Health, Los Angeles, CA USA; 4 Southern California Evidence Review Center, University of Southern California, Los Angeles, CA USA; 5 Veterans Health Administration Greater Los Angeles Healthcare System, Sepulveda, CA USA; 6 Veterans Health Administration, Seattle, WA USA; 7 Veterans Health Administration, Washington, DC USA

**Keywords:** access to healthcare, access management, expert panel, stakeholder engagement, primary care, modified Delphi panel, quality improvement, implementation research

## Abstract

**Objective:**

To identify priorities for improving healthcare organization management of patient access to primary care based on prior evidence and a stakeholder panel.

**Background:**

Studies on healthcare access show its importance for ensuring population health. Few studies show how healthcare organizations can improve access.

**Methods:**

We conducted a modified Delphi stakeholder panel anchored by a systematic review. Panelists (*N* = 20) represented diverse stakeholder groups including patients, providers, policy makers, purchasers, and payers of healthcare services, predominantly from the Veterans Health Administration. A pre-panel survey addressed over 80 aspects of healthcare organization management of access, including defining access management. Panelists discussed survey-based ratings during a 2-day in-person meeting and re-voted afterward. A second panel process focused on each final priority and developed recommendations and suggestions for implementation.

**Results:**

The panel achieved consensus on definitions of optimal access and access management on eight urgent and important priorities for guiding access management improvement, and on 1–3 recommendations per priority. Each recommendation is supported by referenced, panel-approved suggestions for implementation. Priorities address two organizational structure targets (interdisciplinary primary care site leadership; clearly identified group practice management structure); four process improvements (patient telephone access management; contingency staffing; nurse management of demand through care coordination; proactive demand management by optimizing provider visit schedules), and two outcomes (quality of patients’ experiences of access; provider and staff morale). Recommendations and suggestions for implementation, including literature references, are summarized in a panelist-approved, ready-to-use tool.

**Conclusions:**

A stakeholder panel informed by a pre-panel systematic review identified eight action-oriented priorities for improving access and recommendations for implementing each priority. The resulting tool is suitable for guiding the VA and other integrated healthcare delivery organizations in assessing and initiating improvements in access management, and for supporting continued research.

**Electronic supplementary material:**

The online version of this article (10.1007/s11606-019-05541-2) contains supplementary material, which is available to authorized users.

## INTRODUCTION

Providing access to healthcare is a fundamental primary care function and a necessary prerequisite for judging care quality.^1^ Yet, translation of the substantial research on the importance and determinants of access into sustainable access improvement in healthcare delivery organizations has lagged. Few research address the needs of organization leaders and managers for continuous, comprehensive and sustainable access management approaches. Expert panels can provide a basis for moving forward when existing research evidence is insufficient.^2–4^ We initiated a systematic review of research evidence on diverse access management interventions^5^ and a qualitative study of the Veterans Health Administration (VHA) access manager initiative^6^ as part of a 2-year-long project capped by the modified Delphi panel process reported here. Our goal was to provide evidence-informed guidance on top priorities for improving management of patient access to primary care in integrated healthcare systems.

Access is a “wicked” problem.^7, 8^ Wicked problems require solutions that integrate across stakeholder perspectives and span relevant organizational programs and goals. No one solution to a wicked problem will succeed across organizational contexts or across time. All solutions require both trade-offs between alternative goals and ongoing monitoring. We therefore focused on access management as an ongoing organizational activity rather than on access as a once-and-done outcome. We aimed to identify the key organizational structures, processes, and outcomes^9^ that leaders and managers of healthcare delivery systems would need to consider for undertaking access management improvement.

Timely access to primary care is important to integrated healthcare systems, and has been the major focus of access-related performance measurement.^5^ Primary care provides first-line and preventive care, and is at the junction between patients and appropriate use of the wider set of available health services.^10^ The National Academy of Medicine identified timeliness as a fundamental aim for healthcare^11^—and as the least well studied and understood.^12^ However patient needs and preferences are at the foundation of optimal access, and from the patient’s point of view, improved access includes a broader range of considerations other than timeliness, such as availability, accommodation, affordability, and acceptability.^13^

Integration of patient considerations into management strategies is challenging. Patients have diverse perspectives on, for example, accessing primary care team members other than physicians, on group visits, and on non-face-to-face care such as telephone care or secure messaging. Patients may value being seen on a day of their choice, or by their continuity provider, more than they value being seen quickly—or vice versa.^14^ Finally, patients may weigh the effort required to achieve needed access, such as distance to clinic, waiting time, or ease of making appointments, in deciding whether and how to accomplish it.^15, 16^ It is thus not surprising that patient-reported access measures often do not align tightly with timeliness measures.^17–20^ Clearly, improving patient experiences related to access requires integration of patient and system perspectives.

Healthcare system leaders and managers as well as patients face trade-offs in approaching access improvement. The same open access actions that improve access in the short term can lead to increased demand that worsens access in the longer term.^21^ Maximizing timely access and continuity of care can be conflicting goals in the face of fixed primary care resources.^8, 22–24^ In turn, both the supply of services and the demand for them will be influenced by multiple and continuously changing local, regional, and national factors.

In the work presented here, we viewed achieving optimal access as a management challenge within a complex adaptive system framework. In complex adaptive systems, the results of management actions will always be subject to uncertainty^8^ and to unintended consequences;^25, 26^ step by step improvement instructions may therefore not produce the desired result. We aimed instead to use a rigorous evidence base and modified Delphi panel methods to promote the development of targeted access management improvement agendas^27^ by organization leaders and managers, and to promote future access management research. Our objectives were to (1) define access management; (2) identify access management priorities for action; (3) develop recommendations, suggestions for implementation, and references for each priority area; and (4) develop a panel-approved ready to use tool summarizing the results.

## METHODS

### Overview

The study was carried out between January 2016 and May 2018. The stakeholder panel process included data collection and analysis based on in-person panel voting and transcripts as well as five panelist surveys. Key study activities and a project timeline are provided in Table 1 and described below. Other results have been documented elsewhere.^28–30^

**Table 1:** Timeline of Study Activities

Activity	Timing
Collection of access management evidence	1st through 3rd quarter, 2016
Recruitment of a balanced stakeholder panel	4th quarter, 2016
Collection and analysis of pre-panel survey data	4th quarter, 2016
In person stakeholder panel meeting	1st quarter, 2017
Collection and analysis of post-panel survey data	2nd quarter 2017
Development of preliminary evidence-based recommendations for achieving top priorities	3rd and 4th quarters, 2017
Revision of priorities based on panel input	1st quarter, 2018
Development of a tool for use by healthcare organizations	2nd quarter, 2018

The study was assessed by the RAND Human Subjects Protection Committee, found to be of minimal risk, and determined to be exempt. The study was funded by the VHA.

### Collection of Access Management Evidence

Prior to panel activities, we collected reports and manuals focused on how to improve access in VHA^12, 31–33^ or in other major health systems, including Canada’s and Great Britain’s.^25, 26^ We initiated a systematic review of access management by partnering with the Veterans Affairs (VA) Evidence-Based Synthesis Program (ESP).^5^ We also initiated qualitative data collection and analysis of 56 key informant interviews focused on assessing implementation of access-oriented group practice management in VHA (a congressionally mandated initiative).^6^

### Recruitment of a Balanced Stakeholder Panel

Our stakeholder panel included 20 panelists. To identify the 20, we used the “7 P” framework (patients, providers, purchasers, payers, policy makers, product makers, and principal investigators)^34^ informed by an access flow chart and logic model.^30^ We augmented the framework to include representation of expertise in the needs of rural populations; nursing; national, regional, and front-line primary care leadership; large managed care organization policy and practice; a non-U.S. healthcare system (Canada); continuity of care; and access measurement.

### Collection and Analysis of Pre-Panel Survey Data

We developed a literature-based framework of key access management domains (Table 2) and used the framework as the basis for 85 survey items to assess them. The survey asked panelists to rate the priority of addressing each goal as follows: 0 (cannot answer); 1 (very low priority); 2 (low priority); 3 (medium priority); 4 (high priority); or 5 (very high priority, must be addressed this year). We computed reviewer-adjusted (de-biased) means^35^ and the modal value for each item across all panelists. We identified items with substantial disagreement as requiring panel discussion, using a standard deviation (SD) cutoff of more than 0.85. The survey also asked for comments on a preliminary definition of access management.

**Table 2:** Access Management Framework Used for the Pre-Panel Survey

**I. Patient Populations, Service and Practice Contexts**	
A. ***Patient population characteristics*** requiring special access management attention (e.g., difficulty getting to clinic; difficulty using the phone or computer)	
B. ***Primary care practice site*** characteristics requiring special access management attention (e.g., has a rapidly expanding patient population, high demand for walk-in care)	
C. ***Specialty care issues that impact primary care*** (e.g., availability of mental health specialists, availability of specialist care for common problems or for urgent problems)	
**II. Evaluating and Managing Supply and Demand in Primary Care**	
A. ***Primary care practice site level supply and demand context evaluation****:* Parameters that should be routinely measured or evaluated at appropriate intervals, such as annually	
*•****Demand parameters*** (e.g. total number of patients visiting the primary care site during the past year; availability of equipment for enabling access such as telephone or mobile technology)	
*•****Supply parameters*** (e.g., total number of primary care providers versus total number of patients visiting in the past year*;* primary care provider and staff morale)	
B. ***Management approaches to address overall supply and demand mismatches*:** Access managers routinely use or can demonstrate systematic approaches to mismatch (e.g., building capacity ahead of demand; ensuring adequate availability of contingency staffing)	
C*.****Evaluating supply and demand among individual providers and their panels*** (e.g., proportion of all slots for a given clinic day that remain un-booked at the beginning of that clinic day; match provider hours committed to clinical primary care to the clinic sessions actually booked over the past month)	
D. ***Managing supply and demand among individual providers and their panels within a primary care practice site*** (e.g. team RNs prospectively manage demand by leading care coordination for panels; patients can book visits online)	
E. ***Evaluating the availability of alternatives to face-to-face visits at a primary care practice site*** (e.g., weekend, early morning or after hours face to face visits; group visits for common chronic conditions)	
F. ***General management strategies for optimizing the quality of site level patient access*** (e.g. engage patient representatives in strategic planning and improvement projects; ensure inter-professional leader communication and input into decisions)	
**III. Promoting Successful Group Practice Management:** Promising organizational features for promoting access management success (e.g., a clearly identified group practice management structure; ongoing multilevel training of executive, middle-management, front-line staff/clinicians)	
**IV. Managing Demand by Identifying and Managing Complex or Challenging Patients** (e.g., identify the proportions of complex patients in each panel within a site; have mechanisms for engagement of inter-professional input to support primary care provider management of challenging patients, such as disruptive, combative, or severely non-adherent individuals)	
**V. Evaluation of Access Management Short, Medium and Long-Term Outcomes** (e.g., overall time patients must wait for a requested face to face visit, quality of patient experience of access)	

### In-Person Stakeholder Panel Meeting

Panelists attended a 2-day in-person meeting. Panel procedures adhered to consensus methods principles: anonymity (private ranking or voting to avoid dominance group processes); iteration (multiple rounds to allow panelists to change their opinions after discussions); controlled feedback (feedback of the group response); and statistical group response (central tendency and dispersion measures).^36^ We recorded, transcribed, and qualitatively analyzed the in-person panel discussions, including written comments.

We held five simultaneous subpanels^37^ of 6–7 participants in a 1.5-h breakout session during the panel meeting to consider definitions of access and refine definitions of access management.^38^ Subpanels included 14 guest experts in addition to panelists. Each subpanel presented their conclusions to the entire group.^30^

### Collection of Post-Panel Survey Data

After the panel meeting, we used anonymous voting results and formal qualitative content analysis of panel transcriptions to develop revised definitions of access management. We also administered a post-panel survey to all panelists. The survey re-rated pre-panel survey high-priority items and considered new potential high-priority items based on the panel meeting. We included the 10 items from the pre-panel survey with an adjusted mean exceeding 3.75 and for which more than 50% of panelists rated the item as “very high priority.” We analyzed panel discussion transcripts, study team notes, and anonymous votes held during the meeting to identify potential item refinements and potential new panelist-suggested high-priority items. We divided the resulting 14 candidate items into structure, process, and outcome goals, following the Donabedian model,^9^ and asked panelists to rate whether improvement toward this goal was (a) important and urgent (i.e., should be undertaken this year); (b) important but not urgent; or (c) not important. We included all post-panel survey items rated by at least 50% of panelists as important and urgent in a final list of key priorities for action.

### Development of Preliminary Evidence-Based Recommendations for Achieving Key Priorities

To develop recommendations, the study team, supported by an evidence-based practice center, used snowball queries and online searches^30^ to collect additional literature, including gray literature, relevant to each key priority for action. We also electronically searched the transcripts of panel discussions and the studies in the prior access management systematic review^5^ using terms related to each key priority. Based on these results, the study team developed preliminary recommendations and suggestions for implementation for each key priority, with references indicating the basis for each.

### Revision of Priorities Based on Panelist Input

A subset of 14 of the 20 original panelists agreed to participate in reviewing and revising recommendations and suggestions for implementation prior to and during each of 2 conference calls. The 14 panelists represented 6 of the original 7 Ps (patients, providers, purchasers, payers, policy makers, product makers). The 6 declining panelists indicated their willingness to review final materials but did not wish to commit to the additional 2 calls and associated review work.

After each call, the study team used detailed notes, results of anonymous voting, and written comments to revise the recommendations. The first pre-panel call survey asked the 14 panelists whether each proposed recommendation should be included or omitted. The second pre-panel survey asked the 14 to indicate, for each included recommendation, whether the recommendation as written was acceptable or not, and to add any comments. The study team incorporated the final set of priorities, recommendations, suggestions for implementation, and references along with the revised access management definitions in a summary sent to all 20 original panelists for review and comments.

### Development of a Tool for Use by Healthcare Organizations

A project objective was to promote development of improvement agendas^27^ by executive, middle, and frontline managers. To achieve this objective, the project team developed two ready-to-use tools based on final panel results.

## RESULTS

### Stakeholder Panelist Participation

Panelists were primarily associated with VHA, but included one from Canada, one from Kaiser Permanente, and a senior adviser to the US Government Centers for Medicare & Medicaid Services who is also affiliated with the VHA. Panelists represented all pre-identified target 7-P groups and areas of expertise, including two patients.

All panelists (*N* = 20) completed the pre-panel survey. The in-person panel was attended by 19 panelists. Responses from 17 panelists were available for the post-panel survey analysis (response rate 85%). All 14 panelists who agreed to work with the study team to develop recommendations and implementation suggestions, including the two patient representatives, completed participation in at least one of the 2 planned calls. All 20 panelists received final study products for review and comments.

### Panel Consensus on definitions of Access Management

Analysis of access management definitions discussions showed recurrent themes across subpanels. These were timeliness, ease of access, actual versus perceived needs, and importance of the patient perception of access.^28, 30^ Panel members indicated that their final definitions, shown in Table 3, should be viewed as broad guides.

**Table 3:** Final Consensus Definitions of Access Management, Optimal Access Management, and Optimal Access

Concept	Definition
Access management	*Access management* encompasses the set of goals, evaluations, actions and resources needed to achieve patient centered healthcare services that maximize access for defined eligible populations of patients.
Optimal access management	*Optimal access management* engages patients, providers, and teams in continuously improving care design and delivery in order to achieve optimal access.
Optimal access	*Optimal access* balances considerations of equity, patient preferences, patient needs, provider and staff needs, and value.

### Panel Consensus on Priorities for Action

The post-panel survey established eight items as key priorities for action that were both important and urgent (Table 4). Among the eight priorities, panelists identified two key organizational structure targets, four process improvement targets, and two outcome targets.

**Table 4:** Final Eight Key Priorities for Action for Initiating Access Management Improvement

Organizational structure targets	
*○ Interdisciplinary primary care site leadership*: Identify physician, registered nurse, and administrative leaders for each primary care practice site with authority to support access management priorities within local site contexts.	
*○ Clearly identified group practice management structure*: Develop a clearly identified group practice management structure with a designated group practice manager who reports to executive leadership, communicates with individual primary care sites, and can collaborate across roles and service lines (e.g., medicine, nursing, administration).	
Process improvement targets	
*○ Patient telephone access management*: Routinely evaluate the degree to which patient telephone calls are a) answered promptly and b) routed accurately and appropriately, as judged in terms of patients’ clinical needs and preferences.	
*○ Contingency staffing*: Maximize access managers’ routine use or ability to demonstrate systematic approaches to ensuring adequate availability of contingency staffing (i.e., planned minimal excess staffing to cover routine absences (e.g., due to hiring gaps, vacations, illness).	
*○ Nurse management of demand through care coordination*: Maximize primary care team’s registered nurses’ ability to prospectively manage demand by leading care coordination for their panels.	
*○ Proactive demand management by optimizing provider visit schedules*: Maximize primary care team members’ ability to proactively manage demand (e.g., alerts, reminders, and telephone contacts from patients on their panels) by optimizing provider visit schedules (e.g., through triage, prospective “scrubbing” of appointments) to the extent appropriate given their training/licenses.	
Outcome targets	
*○ Quality of patients’ experiences of access:* Assess the quality of the patient’s experience of access (i.e., patient-rated access). We expect patient ratings to reflect both in-person and non-face-to-face (e.g., telephone, secure messaging) care.	
*○ Provider and staff morale in relationship to supply-demand mis-match:* Assess primary care provider and staff morale (e.g., low/high burnout, job satisfaction, or turnover rates) in relation to access mismatch (e.g., panels exceeding recommended size, primary care provider vacancies).	

The panel endorsement of the eight priorities ranged from 53% of panelists (optimizing provider visit schedules; provider and staff morale related to access mismatch) to 100% endorsement (patient telephone access management) (see Table 5). Many dropped items were identified as important but not urgent, such as wait times, use of a variety of non-face to face modalities other than telephone, and evaluation of patient “walk-in” visits. Panelists viewed these as contributing factors, rather than as fundamental priorities.

**Table 5:** Level of Agreement on the Final Eight Key Priorities for Action to Improve Access Management

	*N*	% of responding panelists agreeing with the statement:		
		“Improvement toward this goal is important and urgent”	“Improvement toward this goal is important but not urgent”	“Improvement toward this goal is not important”
Organizational structure targets				
Clearly identified group practice management structure	17	76	24	0
Interdisciplinary primary care site leadership	17	71	29	0
Process improvement targets				
Patient telephone access management	16	100	0	0
Contingency staffing	17	65	29	6
Nurse management of demand through care coordination	17	65	35	0
Proactive demand management by optimizing provider visit schedules	17	53	41	6
Outcome targets				
Quality of patients’ experiences of access	17	88	12	0
Provider and staff morale in relationship to access mismatch	17	53	47	0

### Panel Consensus on Recommendations and Implementation Suggestions

The project team identified 25 potential recommendations, or 2 to 9 per priority area, based on literature review and panel discussions. The total number of recommendations dropped to 14 as panelists came to consensus on those that were most important, and others that could be eliminated because they were duplicative or low impact.

### Final User-Ready Tool for Developing an Access Management Improvement Agenda

For preparing a final tool, panelists directed the team to indicate at what level of an organization endorsement for initiating the recommendations would be required. They also endorsed listing key references for recommendations. Online Appendix 1 includes the final set of key priorities, recommendations for achieving them, and suggestions for implementation, as revised and reviewed by panelists. Online Appendix 2 includes additional introductory material on how to use the tool to develop an access improvement agenda in a “slidedoc”^39^ format (i.e., slides meant to be read, not presented). For example, health systems should consider which key priorities have already been achieved as part of agenda development. A PowerPoint© slidedoc version with additional embedded material is available from the authors.

## DISCUSSION

While access to healthcare has always been of paramount importance, responsibility for ensuring access has often been dispersed across many organizations, leaving none accountable overall. Integrated healthcare organizations such as the VHA and others, however, have both responsibility for enabling access to needed care of all types and population-based data for monitoring it. These circumstances make access challenges more visible and create opportunities for improvement. We initiated a formal systematic review of prior literature and a qualitative study of the experiences of VHA access managers to identify factors influencing access. We then engaged a broadly representative, evidence-informed stakeholder panel in a modified Delphi panel process to identify priorities for action based on their ratings of over 80 of these factors. The resulting eight priorities focus on structure, processes, and outcomes and address essential organizational access management targets. These priorities provide valid current guidance on access management improvement for healthcare organization managers and leaders.

Learning organizations aim to use evidence as the basis for improvement, yet decades of research on access provide limited assistance to healthcare organization leaders and managers as they confront ongoing access challenges.^40^ The inevitability of continuous local and organizational changes in supply and demand, and of the need to integrate patient, provider team, and organizational considerations and preferences, precludes a one-size-fits-all approach. We therefore sought to develop a basis for improvement rather than a set of mandates, and to focus on North American integrated healthcare systems as our context. We think aspects of our work, however, can serve as a foundation for similar initiatives within other organizational contexts, such as practice networks or community-based improvement efforts,

Our systematic review found no studies addressing access as a “wicked” management and policy problem that must be addressed across organizational programs and boundaries.^7^ Most access management improvement interventions in our review focused only on the open access approach.^5^ Open or advanced access focuses on how to manage primary care visit schedules so that a patient is offered a prompt appointment whatever the reason for the visit request.^21^ Existing studies show that system improvement based on single access elements (such as open access^21^) is not only insufficient, but can result in negative consequences. Panelists viewed open access principles as methods for achieving the more fundamental priorities of better group practice management, care coordination, proactive demand management, and patient and provider experience (see recommendations, suggestions, and references for priorities 2, 4, and 6 in Online Appendix 1 focusing on open access) rather than as priority goals in themselves. Within this more comprehensive context, health systems could enlist open access^24, 41, 42^ alongside other relevant approaches as part of an overall access management improvement agenda.

Developing an agreed-upon implementation agenda, spanning organizational boundaries and taking account of local resources, have been shown to be associated with quality improvement initiative success.^27^ The approach described here incorporates each of these features. Our access management tools (Online Appendices 1 and 2) provide concrete guidance on shaping agenda development and provide (Online Appendix 2) an approach for integrating research evidence, local data, and relevant stakeholder input. The priorities themselves span both professions (e.g., nurse, physician, administration) and organizational units (e.g., call centers, information technology, contracting).

In the last decade, many healthcare organizations have changed their access management policies;^43^ most of these changes, however, have not been rigorously evaluated.^5, 17, 18^ Modified Delphi expert panel methods that take account of existing knowledge, while supplementing it with consensus across diverse perspectives, can provide valuable guidance for bridging gaps in research-based knowledge.^2, 3, 34^ A future study testing the effectiveness of achieving adequate performance across all eight priorities identified here, however, would be valuable.

All eight top priorities resulting from the panel met our criterion of endorsement by more than half of the panelists as both important and urgent. The exact level of agreement, however, varied. Interestingly, the most agreed-upon priority (100% agreement) was one that receives scant mention in access literature—i.e., the need for “routine evaluation of the degree to which patient telephone calls are (a) answered promptly and (b) routed accurately and appropriately, as judged in terms of patients’ clinical needs and preferences.” The high level of panelist agreement in the absence of available research strongly suggests a need for additional investigation.

As structure improvement targets, the panel identified interdisciplinary leadership at the local practice site level, with shared governance across physician, nurse, and administrative lines, and achievement of a clear group practice management structure originating at an executive level as top priorities. These targets reflected approaches for achieving the level of boundary spanning communication and decision-making across disciplines and programs required for optimal access management.

As process improvement targets, the panel identified two little-studied influences on access (high-quality telephone access, contingency staffing) and two more commonly referenced in open access and other literature (care coordination and optimizing provider visit schedules).

All panelists, with strong endorsement from the two patient representatives, saw telephone access as fundamental for ensuring appropriate patient safety, scheduling, and coordination.^33^ While virtual or computer access is increasingly important (see priority 7, and suggestions related to priorities 3, 5, 7, and 8, Online Appendix 1), and can reduce patient and provider telephone burden, patient computer abilities and access vary, as do the abilities of provider teams to respond promptly to computer messages. For these and other reasons, computers cannot eliminate the need for high-quality telephone access. Yet healthcare literature provides sparse guidance on how primary care practices should structure or evaluate telephone services. Similarly, panelists identified contingency staffing as critical but often overlooked. They noted that without contingency staffing availability, delivery systems would either fail to deliver adequate access or would be continuously (and likely unsustainably) overstaffed.

In terms of outcomes, panelists viewed both patient and provider experiences of access as critical and intertwined. Because patient experiences of access can reflect all access modalities (e.g., telephone, online, and in-person), panelists judged improving patient experience to be the best overall outcome target for access improvement.

A theme among the panel priorities was the importance of nurse role development both as organizational leaders and as clinical care team leaders. Achievement of optimal nursing roles, as shown in panel recommendations (Online Appendix 1), will require training and role development.

We aimed to provide guidance to health systems, rather than individual primary care practices. However, we expect that, although the relative urgency and importance of the priorities may differ for smaller or unaffiliated practices, all eight priorities are potentially relevant even at the individual practice level. Smaller systems or practices could also consult the overall framework we provide (Table 2) or the survey based on it.^30^

Our study had limitations. While our stakeholders were diverse in professional backgrounds, they represented only large managed care systems in North America. More than half of the panelists had VHA backgrounds. During panel discussions, however, non-VHA panelists re-iterated the extent to which priorities were common across delivery systems, differing mostly in the extent to which a priority was currently problematic or had already been adequately achieved. Additionally, our panel was designed to include only 20 participants. This size is maximal for an in-person modified Delphi panel. A future online larger panel may be helpful for addressing other contexts.^37^ As another limitation, we did not achieve (and did not aim to achieve) full agreement among panelists; we followed strict procedures to identify rather than coerce consensus. However, disagreements on priorities were largely limited to whether a priority was both urgent and important, or simply important, and all participating panelists endorsed our final definitions, priorities, recommendations, and suggestions. We also did not assess the costs or methods for paying for enhanced access. Finally, our results are intended to be formative rather than definitive.

Our findings imply that the current healthcare organization focus on timeliness of access and on achieving open access goals is too narrow to succeed. Because it is often the factor upon which achievement of all others may depend, we recommend establishing cross-cutting access management (see structure-related priorities) as a starting point. We then recommend a formal process of assessing current accomplishments in each priority area and engaging stakeholders in addressing one or two (Online Appendix 2).

In summary, this study provides a valid foundation for action for achieving optimal access management within healthcare organizations, as well as providing a basis for future research. Our final eight key priorities are concise action points and are linked to relevant evidence and guidance on implementation in ready-to-use tools (Appendices 1 and 2). Our work as reported here can provide healthcare organization leaders and managers, particularly those in integrated healthcare organizations, with a foundation for undertaking access management improvement, while tailoring it to their own contexts.

## Electronic supplementary material

ESM 1 (DOCX 48 kb)

ESM 2 (DOCX 6279 kb)
