# Supplementary material for: Eight Priorities for Improving Primary Care Access Management in Healthcare Organizations: Results of a Modified Delphi Stakeholder Panel
Source: J Gen Intern Med. 2019 Nov 14;35(2):523–30. doi: 10.1007/s11606-019-05541-2 (PMC7018673; doi:10.1007/s11606-019-05541-2)
Supplement: Supplementary file 1 — (DOCX 48 kb) [file 11606_2019_5541_MOESM1_ESM.docx]

**Appendix 1. Recommendations, Suggestions for Implementation, and References for Priority Actions***

| Organizational Structure Targets | |
| --- | --- |
| **Priority Action #1** | **Identification of Primary Care Practice Site Leaders** |
|  | *Identify physician, registered nurse, and administrative leaders for each primary care practice site with authority to support access management priorities within local site contexts.* |
| **Recommendation 1.A**  *Initiation Level*: Must be initiated at the executive level | **Ensure that the inter-professional structure of team-based care models, such as the patient-centered medical home, is reflected in strong integrated inter-professional governance at the primary care practice site level, including explicitly identified site-level physician, RN, and administrative leaders.** |
| **Suggestions for implementing the recommendation** | - Consider models of shared governance at the local level. Shared governance is a system of management that aims to empower staff in decision-making at the local unit level, such as at the primary care practice level, with the goal of enabling health care professionals to develop multi-professional care. In the United States, much of the shared governance focus has been on nurses as participants in shared governance, often in hospitals. In the United Kingdom, there has been significant focus on primary care, and on the role of administrative managers. There are few empirical quantitative outcome studies on these models; however, there is substantial qualitative evaluation information supporting them. - Ensure strong central (executive) leadership engagement, including allocation of resources, such as staff time, in implementing a shared governance model; without this, very autonomous practices without a prior shared governance culture are unlikely to succeed. - Assess and improve primary care practice leadership team communication by developing willingness to participate in shared decision-making and ability to focus on task conflict and differences in opinion rather than on personality differences and interpersonal tensions. - Avoid top-down pressure to deliver rapid measurable changes, lack of attention to professional autonomy, and a focus on blame. These are major impediments to success (balance with standardization). - Promote open accountability, willingness to learn across disciplines or professions and from mistakes, and willingness to problem-solve through quality improvement. These characteristics are linked to the concept of psychological safety, which is predictive of success. - Legitimize and balance managerial (administrative) and health care professional (physicians, nurses, and others) perspectives; respect for professional autonomy and for the benefits of shared administrative approaches are essential. - Develop approaches for sharing learning across primary care practices; this can facilitate effective local practice site leadership. |
| **References and further reading for the recommendation** | Campbell, S. M., R. Sheaff, B. Sibbald, M. N. Marshall, S. Pickard, L. Gask, S. Halliwell, A. Rogers, and M. O. Roland, “Implementing Clinical Governance in English Primary Care Groups/Trusts: Reconciling Quality Improvement and Quality Assurance,” *Quality & Safety in Health Care*, Vol. 11, No. 1, March 2002, pp. 9–14.  Clavelle, J. T., T. Porter O’Grady, M. J. Weston, and J. A. Verran, “Evolution of Structural Empowerment: Moving from Shared to Professional Governance,” *Journal of Nursing Administration*, Vol. 46, No. 6, June 2016, pp. 308–312.  Grant, S., A. Ring, M. Gabbay, B. Guthrie, G. McLean, F. S. Mair, G. Watt, D. Heaney, and C. O’Donnell, “Soft Governance, Restratification and the 2004 General Medical Services Contract: The Case of UK Primary Care Organisations and General Practice Teams,” *Sociology of Health and Illness*, Vol. 37, No. 1, January 2015, pp. 30–51.  King’s Fund Commission on Leadership and Management in the NHS, *The Future of Leadership and Management in the NHS: No More Heroes*, London: The King’s Fund, 2011.  Kutney-Lee, A., H. Germack, L. Hatfield, S. Kelly, P. Maguire, A. Dierkes, M. Del Guidice, and L. H. Aiken, “Nurse Engagement in Shared Governance and Patient and Nurse Outcomes,” *Journal of Nursing Administration*, Vol. 46, No. 11, November 2016, pp. 605–612.  Marshall, M., R. Sheaff, A. Rogers, S. Campbell, S. Halliwell, S. Pickard, B. Sibbald, and M. Roland, “A Qualitative Study of the Cultural Changes in Primary Care Organisations Needed to Implement Clinical Governance,” *British Journal of General Practice*, Vol. 52, No. 481, August 2002, pp. 641–645.  Solberg, L. I., M. C. Hroscikoski, J. M. Sperl-Hillen, P. J. O’Connor, and B. F. Crabtree, “Key Issues in Transforming Health Care Organizations for Quality: The Case of Advanced Access,” *Joint Commission Journal on Quality and Safety*, Vol. 30, No. 1, January 2004, pp. 15–24.  Sullivan, E. E., Z. Ibrahim, A. L. Ellner, and L. J. Giesen, “Management Lessons for High-Functioning Primary Care Teams,” *Journal of Healthcare Management*, Vol. 61, No. 6, November/December 2016, pp. 449–465. |
|  |  |
| **Recommendation I.B**  *Initiation Level:* Can be initiated by midlevel managers, including unit managers, primary care site leaders, or care team leaders | **Ensure that inter-professional primary care practice leadership is trained in problem-solving approaches and in how to teach these to providers and staff.** |
| **Suggestions for implementing the recommendation** | - Teach the teachers (practice site leaders) to use and transmit the problem-solving approaches typical of high-functioning teams, including, for example, practice redesign and quality improvement techniques. - Develop organizational strategies that facilitate success for local quality improvement innovation. |
| **References and further reading for the recommendation** | Kim, L. Y., D. E. Rose, L. M. Soban, S. E. Stockdale, L. S. Meredith, S. T. Edwards, C. D. Helfrich, and L. V. Rubenstein, “Primary Care Tasks Associated with Provider Burnout: Findings from a Veterans Health Administration Survey,” *Journal of General Internal Medicine*, Vol. 33, No. 1, January 2018, pp. 50–56.  Sullivan, E. E., Z. Ibrahim, A. L. Ellner, and L. J. Giesen, “Management Lessons for High-Functioning Primary Care Teams,” *Journal of Healthcare Management*, Vol. 61, No. 6, November/December 2016, pp. 449–465.  Wu, F. M., L. V. Rubenstein, and J. Yoon, “Team Functioning as a Predictor of Patient Outcomes in Early Medical Home Implementation,” *Health Care Management Review*, Vol. 43, No. 3, July 2018, pp. 238–248. |
| **Priority Action #2** | **Group Practice Management Structure** |
|  | *Develop a clearly identified group practice management structure with a designated group practice manager who reports to executive leadership, communicates with individual primary care sites, and can collaborate across roles and service lines (e.g., medicine, nursing, administration).* |
| **Recommendation 2.A**  *Initiation Level*: Must be initiated at the executive level | **Identify a group practice manager position at a high enough level to report effectively to executive leadership while connecting executive leadership and relevant middle managers with frontline primary care practice–level access-related activities and concerns.** |
| **Suggestions for implementing the recommendation** | **Characteristics of successful group practice managers (GPMs) include that the GPM:**   - serves as the point person for helping local primary care site leaders respond effectively to both central and frontline demands - reports to the executive team, participates in organizational strategic planning, and has sufficient authority to implement needed improvements - has both good people skills and data skills, and uses them to enable and monitor access improvement while also managing expectations - has oversight for the access management program, including identifying gaps and establishing processes to close them. |
| **References and further reading for the recommendation** | King’s Fund Commission on Leadership and Management in the NHS, *The Future of Leadership and Management in the NHS: No More Heroes*, London: The King’s Fund, 2011.  Murphy, Janet, and Acting Deputy Under Secretary for Health for Operations and Management, “Implementation of Standardized Position Description for Ambulatory Care Operations Director, Group Practice Manager,” memorandum, Washington, D.C.: U.S. Department of Veterans Affairs, 2015.  Patient Aligned Care Team, *The Path to Open Access: A Primary Care Roadmap*, Washington, D.C., February 2016.  Solberg, L. I., M. C. Hroscikoski, J. M. Sperl-Hillen, P. J. O’Connor, and B. F. Crabtree, “Key Issues in Transforming Health Care Organizations for Quality: The Case of Advanced Access,” *Joint Commission Journal on Quality and Safety*, Vol. 30, No. 1, January 2004, pp. 15–24.  Sullivan, E. E., Z. Ibrahim, A. L. Ellner, and L. J. Giesen, “Management Lessons for High-Functioning Primary Care Teams,” *Journal of Healthcare Management*, Vol. 61, No. 6, November/December 2016, pp. 449–465.  Veterans Health Administration, *Outpatient Scheduling Processes and Procedures*, VHA Directive 1230, Washington, D.C.: U.S. Department of Veterans Affairs, 2016. |
| **Recommendation 2.B**  *Initiation Level*: Can be initiated by midlevel managers, including unit managers, primary care site leaders, or care team leaders | **Identify and document the specifics of a group practice management program and team, including key stakeholders in the team’s success, and ensure that the team is trained in (a) how to promote stakeholder engagement, including frontline staff, and (b) how to access and use data for improvement.** |
| **Suggestions for implementing the recommendation** | - Create an orchestrated access management team in each facility. - Incentivize clinicians and staff members to work in teams to address the issue of hierarchy, which limits sharing of information and experience; clarify roles. Engage team members by providing feedback on improvement measures and how changes are improving the situation for patients. - Incorporate flexibility into the access management program to be able to customize the program to unique site and patient needs. - Create a group practice management culture that is goal-directed rather than punitive and that focuses on improvement. - Identify or develop tools to track and manage patient care, and use the tools to inform executive management. - Expect and overcome resistance when decision-making crosses professional silos; build psychological safety into improvement efforts to address risk aversion; promote a culture of problem-solving. - Display GPM-gathered data in one place (e.g., a dashboard) where the data can be easily reviewed by the GPM and shared with others (e.g., clinic leaders) to identify and solve problems. - Ensure that GPM-gathered data are clean and accurate and reflect what is being done to facilitate access to care and enable decision-making. - Training for group practice managers is critical, particularly regarding acquiring, utilizing, and communicating data. - Engage team members by providing feedback on improvement measures and how changes are improving the situation for patients. - Engage all team members regardless of hierarchy in understanding the value of data generation and use in improvement and change programs. - Ensure that the GPM team is trained in advanced access techniques. - Train GPMs in inter-professional shared decision-making, such as shared governance (see Recommendation #1. A, on the identification of primary care practice site leaders). |
| **References and further reading for the recommendation** | Kim, L. Y., D. E. Rose, L. M. Soban, S. E. Stockdale, L. S. Meredith, S. T. Edwards, C. D. Helfrich, and L. V. Rubenstein, “Primary Care Tasks Associated with Provider Burnout: Findings from a Veterans Health Administration Survey,” *Journal of General* *Internal Medicine*, Vol. 33, No. 1, January 2018, pp. 50–56.  LeRouge, C., Savitha Sangameswaran, Bianca Frogner, Cyndy Snyder, Lisa Rubenstein, Susan Kirsh, and George Sayre, “The Group Practice Manager in the VHA: A View from the Field,” forthcoming.  NHS Practice Management Network, *Improving Access, Responding to Patients: A “How-To” Guide for GP Practices*, London: UK Department of Health, June 2009.  Patient Aligned Care Team, *The Path to Open Access: A Primary Care Roadmap*, Washington, D.C., February 2016.  Sullivan, E. E., Z. Ibrahim, A. L. Ellner, and L. J. Giesen, “Management Lessons for High-Functioning Primary Care Teams,” *Journal of Healthcare Management*, Vol. 61, No. 6, November/December 2016, pp. 449–465. |
|  |  |
| Process Improvement Targets | |
| **Priority Action #3** | **Telephone Access**  *Routinely evaluate the degree to which patient telephone calls are (a) answered promptly and (b) routed accurately and appropriately, as judged in terms of patients’ clinical needs and preferences.* |
| **Recommendation 3.A**  *Initiation Level*: Must be initiated at the executive level | **Ensure that the set of telephone management metrics incorporates four key principles:**   - following calls to resolution - accounting for both call center and primary care team roles in telephone management, promoting substitution of telephone management for in-person management whenever appropriate - promoting metric availability at local site levels to support targeted telephone management improvement initiatives - incorporating patient preferences and experiences into metric development and interpretation. |
| **Suggestions for implementing the recommendation** | **Suggested call center measures include:**   - the average speed of answer by the call center for all inbound calls (e.g., the percentage of calls answered within 60 seconds) - whether calls are answered more quickly at certain times of day versus others - 24-hour availability of call answering - measures beyond electronic data (e.g., record call center calls for quality assessment) - call-back workload (e.g., the number of call-back attempts to patients per 100 initiating calls from the patient or call center) - provider satisfaction (e.g., with types of appointments booked through the telephone, and reasons for dissatisfaction; timeliness of patient appointments in the community-based programs).   **Suggested primary care team measures include:**   - average speed of answer to the patient from the final call destination for calls routed from a call center to a medical office or clinic - number or proportion of potentially avoidable calls (e.g., percentage of patients who call and leave more than one message for the clinic before being able to schedule an appointment; clinician assessments of when and what types of calls could be handled another way) - average speed of primary care team answers to (a) all inbound calls and recorded messages from the call center, and (b) all inbound patient calls.   **Suggested first call to resolution measures include:**   - measures beyond electronic data (e.g., use provider quality review of a set of medical records to determine whether calls are being routed accurately and appropriately based on the patients’ clinical needs and preferences; survey patients’ satisfaction with their ability to contact their physician or care delivery team with a medical question [see Recommendations #7.A, B on patient access experience) - measures of local primary care site call resolution to provide individual sites with data for improvement - rates of multiple patient call-backs (e.g., the percentage of patients resolving their problem on a first call, the percentage of patients making a second call, the percentage of patients lost to follow-up after leaving a message, the percentage of patients leaving more than one message before achieving resolution). - **Suggested patient access experience measures** (see Recommendations 7.A, B on patient access experience) |
| **References and further reading for the recommendation** | Bowman, B. and S. Smith (2010). "Primary Care DirectConnect: How the Marriage of Call Center Technology and the EMR Brought Dramatic Results-A Service Quality Improvement Study." Perm J 14(2): 18-24.  Trustees of Dartmouth College (2002). Improving Patient Access to Care. Hanover, NH, Dartmouth College. |
| **Recommendation 3.B**  *Initiation Level*: Can be initiated by midlevel managers, including unit managers, primary care site leaders, or care team leaders | **Ensure that appropriate telephone management strategies are focused on four key principles:**   - enabling links to continuity primary care teams whenever possible and appropriate - enabling telephone calls to access available non–primary care resources appropriately, comprehensively, and accurately - facilitating local health care system and site improvement based on data - using best available technology for incorporating patient preferences into telephone answering protocols. |
| **Suggestions for implementing the recommendation** | **Suggestions to facilitate telephone management contacts include:**   - Identify and document all acceptable methods for linking patient calls to their primary care teams and providers. - Identify all resources that are accessible by telephone and make available as appropriate to staff and patients. - Develop a plan for regularly updating relevant health resource contact information.   **Suggestions to support ongoing, broad-based telephone management improvements include:**   - Set improvement goals for telephone answering services by engaging key stakeholders, including patients. - Engage local stakeholders by sharing metrics results widely (e.g., report them in executive leadership and other staff meetings; post on websites or in newsletters) and celebrating improvements. - Identify approaches and mechanisms for initiating improvement initiatives.   **Suggestions for use of technology to incorporate patient preferences in call center protocols include:**   - Inform patients on hold about estimated wait times, and give the patient the option of waiting longer or leaving a message. - Use different approaches for patients requesting a medical advice contact versus appointment scheduling contact. - Remove such messages as “Voicemail is full; call back another time” from call center or designated primary care site phones. - Identify key areas in which patient preferences should guide the call center’s response (e.g., when the call is not emergent and the patient prefers a call or appointment with his or her assigned provider or team rather than a quicker appointment or answer from an alternative) and train call center staff accordingly. - Remove performance or technological barriers to responding appropriately to patient preferences. - Set improvement goals to increase the frequency of real-time medical advice provided to patients with a medical question by a known and trusted caregiver. - Explore technology to connect the call center to the electronic medical records so that incoming calls can be automatically assessed for medical questions and automatically routed directly to the patients’ physician or care team. |
| **References and further reading for the recommendation** | Bowman, B. and S. Smith (2010). "Primary Care DirectConnect: How the Marriage of Call Center Technology and the EMR Brought Dramatic Results-A Service Quality Improvement Study." Perm J 14(2): 18-24.  Bunik M, Glazner JE, Chandramouli V, Emsermann CB, Hegarty T, Kempe A. Pediatric Telephone Call Centers: How Do They Affect Health Care Use and Costs? Pediatrics. 2007;119(2):e305-e313.  NHS Practice Management Network, Improving Access, Responding to Patients: A “How-To” Guide for GP Practices, London: UK Department of Health, June 2009.  Stacey D, Graham ID, O'Connor AM, Pomey MP. Barriers and facilitators influencing call center nurses' decision support for callers facing values-sensitive decisions: A mixed methods study. Worldv Evid-Based Nu. 2005;2(4):184-195. |
| **Recommendation 3.C**  *Initiation Level*: Can be initiated by midlevel managers, including unit managers, primary care site leaders, or care team leaders | **Ensure that telephone management initiatives target call center and primary care team staff satisfaction and stability by prioritizing:**   - appropriate staffing - incentives for high-quality performance. |
| **Suggestions for implementing the recommendation** | **Suggestions for call center staffing strategies include:**   - Assess overall call center staffing and turnover relative to call volumes and “right size” staffing, even if it means limiting the number of practices or clinics served by the call center at a given time. - Ensure increased call center staff availability during typical high-volume call times. - Incentivize call center personnel (e.g., ensure appropriate General Services levels for level of responsibility; reward high-quality performance; ensure pathways for advancement). - Reduce friction and increase positive interactions between call center staff and clinical care teams (e.g., schedule meetings between the call center director and the care teams as needed to get buy-in for call center procedures).   **Suggestions for primary care team staffing strategies include:**   - Assess primary care team staffing, satisfaction, and stability in relation to telephone management demand (see Recommendation #6.A on provider experience). - Ensure increased primary care team staff availability for responding to patient calls and call center messages during typical high-volume call times. - Identify standard methods aimed at minimizing demand for telephone access while also minimizing demand for unnecessary in-person visits (e.g., robocall high-volume information, such as flu-shot reminders). |
| **References and further reading for the recommendation** | Health Quality Ontario, “Predict the Expected: Contingency Plans to Manage Advanced Access Schedules,” Toronto, undated.  NHS Practice Management Network, Improving Access, Responding to Patients: A “How-To” Guide for GP Practices, London: UK Department of Health, June 2009. |
| **Recommendation 3.D**  *Initiation Level*: Can be initiated by midlevel managers, primary care site leaders, or care team leaders | **Ensure that patients with hearing loss or difficulty attending face-to-face visits are accommodated through telephone adaptations, print materials, or other virtual technologies.** |
| **Suggestions for implementing the recommendations** | - Identify target vulnerable groups for ensuring telephone access; always include patients with mental health issues and patients with hearing loss. - Assess patient telephone access and care experiences among other vulnerable patient groups seeking care at a given primary care site, such as those who live distant from the site; are homeless; are women veterans; are frail elderly; have language difficulties; or belong to ethnic, cultural, or sexual minorities. Institute training, technology, or protocols for ensuring appropriate and effective communication with clinic staff regarding needed care. - Assess patient satisfaction with telephone access among each target vulnerable group, and institute training, technology, or protocols for improvement where needed. - Ensure prompt availability of local help for mental health problems (e.g., “hot numbers” to primary care and to mental health specialty, or to an appropriately skilled triage, for patients with mental health problems, in addition to national suicide hotlines). - Reach out to patients with mental health diagnoses proactively through a hotline. - Identify patients with critical hearing loss, and proactively identify technology or alternative communication methods to accommodate telephone access |
| **References and further reading for the recommendation** | Berry, L. L., D. Beckham, A. Dettman, and R. Mead, “Toward a Strategy of Patient-Centered Access to Primary Care,” *Mayo Clinic Proceedings*, Vol. 89, No. 10, October 2014, pp. 1406–1415.  NHS Practice Management Network, *Improving Access, Responding to Patients: A “How-To” Guide for GP Practices*, London: UK Department of Health, June 2009 |
| **Priority Action #4** | **Development of Contingency Staffing Approaches**  *Maximize access managers’ routine use or ability to demonstrate systematic approaches to ensuring adequate availability of contingency staffing (i.e., planned minimal excess staffing to cover routine absences, such as those resulting from hiring gaps, vacations, illness).* |
| **Recommendation 4.A**  *Initiation Level*: Must be initiated at the executive level | **Analyze demand data and use that information to anticipate upcoming needs for contingency staffing either from internal staffing resources (e.g., local or regional primary care teams) or from external sources (e.g., contracted temporary staffing). Contingency staffing is a work-force method in which staff members are hired as and when needed for limited periods of time.** |
| **Suggestions for implementing the recommendation** | **Suggested approaches to predicting demand include:**   - Use demand data to predict daily, weekly, and seasonal demand and adjust staffing accordingly. - Add more appointment times for seasonal events, such as seasonal migration from cold climates to warm climates, flu shot clinics during flu season, or pre-school physicals for clinics serving children. - Assess any backlog and consider whether contingency staffing is needed to reduce or eliminate it. - Plan to cover leave times by reviewing past-year leave data. Then calculate the contingency staffing needed to cover leave and arrange for it on a routine basis, such as by hiring or arranging for regular but intermittent additional contingency staff to cover leave times. |
|  | **Suggested approaches to matching contingency staffing supply to demand include:**   - Use daily huddles to review demand and to proactively match daily supply and demand, identifying any upcoming needs for contingency staffing. - Schedule discretionary time for providers to cover absences of other providers or team members. - Minimize the need for contingency staffing through planned management of provider leave time (e.g., hold time in a vacationing provider’s schedule for the first week back from vacation; when the provider leaves, start filling morning appointments of the return week; and when provider returns, start filling afternoon appointments of that week). - Plan for the sudden absence of a provider or other team member—short-term (e.g., sick day) or long-term—by identifying emergency contingency staffing sources (e.g., through community support agencies and care management companies or through shared internal staff across panels). - Minimize the use of external contingency staffing by cross-training team members and internal contingency staff (e.g., float team trained to cover responsibilities throughout the clinic when needed; scheduling staff trained to clean instruments and set up rooms for procedures; nursing staff trained to do scheduling if necessary; scheduling or reception staff trained to gather patient information and assign patients to exam rooms; check-in and check-out staff trained to fill in for each other; physicians, nurse practitioners, and physician assistants trained to substitute for each other). |
| **References and further reading for the recommendation** | Health Quality Ontario, “Predict the Expected: Contingency Plans to Manage Advanced Access Schedules,” Toronto, undated.  Institute for Healthcare Improvement, “Changes: Optimize the Care Team,” webpage, accessed 1/28/2019.  Murray M, Berwick DM. Advanced access: reducing waiting and delays in primary care. JAMA. 2003;289(8):1035-1040.  Steinbauer, J. R., K. Korell, J. Erdin, and S. J. Spann, “Implementing Open-Access Scheduling in an Academic Practice,” Family Practice Management, Vol. 13, No. 3, March 2006, pp. 59–64. |
| **Priority Action #5** | **Nurse management of demand through care coordination Nurse management of demand through care coordination**  *Maximize the ability of the primary care team’s registered nurses to prospectively manage demand by leading care coordination for their panels.* |
| **Recommendation 5.A**  *Initiation Level*: Must be initiated at the executive level | **RNs should be specifically trained and supported to become leaders in proactive demand management and care coordination at three distinct levels (RN executives, RN site-level or multisite-level nurse managers, and RN team-level care coordinators).** |
| **Suggestions for implementing the recommendation** | **Training at each level should include, for example:**   - appropriate leadership skills (e.g., inter-professional communication, understanding access and care coordination, development of RN primary care careers and roles) - basic primary care access and care coordination management and outcome measures, as well as RN roles in promoting access improvement - primary care team functioning and the RN role within it - panel management based on data, such as dashboards or care coordination software - available alternatives to individual in-person visits, such as telehealth, telephone care, secure messaging, group visits, and e-consults - contingency management (see Recommendation #4.A, on development of contingency staffing) - demand management (see Recommendation #8.A, on maximizing the ability to manage demand).   **Support should include:**   - ensuring that RNs have the appropriate level of authority and appropriate time allocation for achieving effective hands-on demand and care coordination management. |
| **References and further reading for the recommendation** | Bodenheimer, T., and L. Bauer, “Rethinking the Primary Care Workforce—An Expanded Role for Nurses,” *New England Journal of Medicine*, Vol. 375, No. 11, September 15, 2016, pp. 1015–1017.  Health Quality Ontario, “Advanced Access and Efficiency Workbook for Primary Care,” Toronto, July 2011.  Health Quality Ontario, “Predict the Expected: Contingency Plans to Manage Advanced Access Schedules,” Toronto, undated.  Institute of Medicine, *Transforming Health Care Scheduling and Access: Getting to Now*, Washington, D.C.: National Academies Press, 2015.  Khan, Phillip J. Longman, Lucretia M. McClenney, Martin R. Steele, Charlene M. Taylor, and Marshall W. Webster, Final Report of the Commission on Care, Washington, D.C.: Commission on Care, June 30, 2016.  King’s Fund Commission on Leadership and Management in the NHS, *The Future of Leadership and Management in the NHS: No More Heroes*, London: The King’s Fund, 2011.  Patient Aligned Care Team National Evaluation and Demonstration Laboratories, “Key Findings About Improving Access from the PACT Demonstration Laboratory Initiative FY10–FY16,” Washington, D.C.: Veterans Health Administration, 2016.  Schlichting, Nancy M., Delos M. Cosgrove, David P. Blom, David W. Gorman et al. Final Report of the Commission on Care, Washington, D.C.: Commission on Care, June 30, 2016. |
|  |  |
| Outcome Targets | |
| **Priority Action #6** | **Provider Experience**  *Assess primary care provider and staff morale (e.g., low/high burnout, job satisfaction, or turnover rates) in relation to access mismatch (e.g., panels exceeding recommended size, primary care provider vacancies).* |
| **Recommendation 6.A**  *Initiation Level:* Must be initiated at the executive level | **Develop a central organizational focus on tracking primary care provider and staff morale (including retention, recruitment) in relation to access mismatch at the local primary care practice, local health care system (medical center), and regional levels.** |
| **Suggestions for implementing the recommendation** | **Assess each primary care practice for supply versus demand mismatch at the site level:**   - Assess overall patient numbers at each primary care practice site, calculated as the patient load (e.g., the number of primary care patients seen at the site + the number on a wait list in the prior year) versus the expected number of patients that currently available providers and staff could see (e.g., the number of full-time employment equivalent providers, teamlets, or patient panels × the number of patients expected to be cared for per provider, teamlet, or panel at the site). If the difference between the expected number of patients that could be seen and the number of patients seen or waiting to be seen last year is substantial, that is a major mismatch that is likely to cause provider and staff burnout. - Ensure that data assessing burnout and turnover in relation to local primary care site supply versus demand mismatch are assessed and communicated at regular intervals to regional and local health care system leadership.   **Use standard measures to assess morale, such as:**   - burnout (e.g., the Maslach Burnout Inventory [MBI], including MBI-Human Services Survey, MBI-Human Services Survey for Medical Personnel, or MBI-General Survey) - job satisfaction (e.g., Areas of Worklife Survey that assesses aspects of work experience; Institute for Healthcare Improvement job satisfaction measure) - provider and staff turnover (e.g., Institute for Healthcare Improvement Measure of Nurse Turnover Rate assessing the number of voluntary uncontrolled separations during the month for unit registered nurses and advanced practice nurses divided by the number of unit employees [full-time + part-time] on the last day of the month for registered nurses and advanced practice nurses, multiplied by 100 to get the percentage).   **Focus on supply-demand mismatch, but assess additional causes of low morale:**   - Assess provider and staff turnover, burnout, and staff satisfaction in relation to changes in access management, such as implementation of advanced access features.   **Focus on both retention and recruitment:**   - Assess current provider and staff retention incentives and whether they are sufficient to retain providers and staff in locations with access mismatch. - Interview departing providers and staff to assess reasons for leaving, categorize causes, and report periodically (e.g., yearly) to leadership. |
| **References and further reading for the recommendation** | Buell, Ryan W., R. S. Huckman, and S. Travers, *Improving Access at VA*, Boston, Mass.: Harvard Business School, November 2016.  Dixon, S., F. C. Sampson, A. O’Cathain, and M. Pickin, “Advanced Access: More Than Just GP Waiting Times?” *Family Practice*, Vol. 23, No. 2, April 2006, pp. 233–239.  Hussey, P.S., J. S. Ringel, S. Ahluwalia, R. A. Price et al. *Resources and Capabilities of the Department of Veterans Affairs to Provide Timely and Accessible Care to Veterans*, Santa Monica, Calif.: RAND Corporation, RR-1165/2-VA, 2015.  Institute for Healthcare Improvement, “Measures: Percent of Voluntary Nurse Turnover,” webpage, undated-b.  Kennedy, J. G., and J. T. Hsu, “Implementation of an Open Access Scheduling System in a Residency Training Program,” Family Medicine, Vol. 35, No. 9, October 2003, pp. 666–670.  Leiter, Michael P., and Christina Maslach, “Areas of Worklife Survey,” Mind Garden, 2000.  Maslach, Christina, and Susan E. Jackson, “MBI—Human Services Survey,” Mind Garden, 1986.  Mehrotra, Ateev, Lori Keehl-Markowitz, and John Z. Ayanian, “Implementation of Open Access Scheduling in Primary Care: A Cautionary Tale,” Annals of Internal Medicine, Vol. 148, No. 12, 2008, pp. 915–922.  Patient Aligned Care Team, The Path to Open Access: A Primary Care Roadmap, Washington, D.C., February 2016.  Pickin, M., A. O’Cathain, F. C. Sampson, and S. Dixon, “Evaluation of Advanced Access in the National Primary Care Collaborative,” *British Journal of General Practice*, Vol. 54, No. 502, May 2004, pp. 334–340.  Pierdon, S., T. Charles, K. McKinley, and L. Myers, “Implementing Advanced Access in a Group Practice Network,” *Family Practice Management*, Vol. 11, No. 5, May 2004, pp. 35–38.  Schlichting, Nancy M., Delos M. Cosgrove, David P. Blom, David W. Gorman, Thomas E. Harvey, Joyce M. Johnson, Ikram U. Khan, Phillip J. Longman, Lucretia M. McClenney, Martin R. Steele, Charlene M. Taylor, and Marshall W. Webster, Final Report of the Commission on Care, Washington, D.C.: Commission on Care, June 30, 2016.  Tantau, C., “Tools: Provider and Staff Satisfaction Survey,” webpage, Tantau & Associates, 2018. |
|  |  |
| **Priority Action #7** | **Patient Access Experience**  *Assess the quality of the patient’s experiences of access (i.e., patient-rated access). We expect patient ratings to reflect both in-person and non-face-to-face (e.g., telehealth, telephone, secure messaging) care* |
| **Recommendation 7.A**  *Initiation Level*: Must be initiated at the executive level | **Regularly assess patient access experiences at the primary care site patient population level, using a reliable and valid survey.** |
| **Suggestions for implementing the recommendation** | **Suggested surveys include:**   - the Supplemental Access Items for the CAHPS^®^ Health Plan Survey 5.0 - Survey of Healthcare Experiences of Patients Consumer Assessment of Health Plans – Patient-Centered Medical Home (SHEP CAHPS PCMH) questions on access.   **Measures of timeliness alone are not sufficient:**   - Assess both urgent/same-day access and routine access for new and established patients. - Measure timeliness of bonded and matched access (e.g., access to the patient’s **Suggestions for implementing the recommendation** assigned site, continuity provider, and team) separately from simple timeliness of access, particularly for nonurgent problems. - Incorporate the hassle factor in surveys of patient access experience. - Consider patient focus group input on existing patient survey designs and what the surveys do and do not reflect. - Consider the access experiences and perceptions of patient family members, who may be carrying much of the access burden for some patients. - Establish a patient and family advisory council for each primary care clinic to provide feedback to improve the patient access experience.   **Recognize the following in interpreting results:**   - The degree to which a visit met patient choice (e.g., whether, for a specific visit, bonding and matching were more important or urgency and timeliness were more important) will ultimately shape overall satisfaction. - Different types of patients (e.g., younger versus older, chronically ill versus healthy) have different priorities, as do patients who are anxious, in pain, missing work, or being affected in terms of other daily living activities. - Non-face-to-face modalities (e.g., telehealth, telephone, or secure messaging visits, particularly with continuity providers or teams) can substitute substantially for in-person visits and affect patient experience measures. |
| **References and further reading for the recommendation** | Agency for Healthcare Research and Quality, “Supplemental Access Items for the CAHPS Health Plan Survey 5.0: Adult,” webpage, June 2016.  Parchman, M. L., P. H. Noel, and S. Lee, “Primary Care Attributes, Health Care System Hassles, and Chronic Illness,” Medical Care, Vol. 43, No. 11, November 2005, pp. 1123–1129. |
| **Recommendation 7.B**  *Initiation Level*: Can be initiated by midlevel managers, including unit managers, primary care site leaders, or care team leaders | **Regularly collect real-time data in primary care sites at the time of visits.** |
| **Suggestions for implementing the recommendation** | **Suggested surveys include:**   - American Association of Family Physicians questionnaire at each patient primary care in-person contact - Health Quality Ontario questionnaire at each patient primary care in-person contact - Institute for Healthcare Improvement and Dartmouth College’s “Today’s Office Visit Survey Card” at each patient primary care in-person contact.   **Suggested additional evaluations:**   - Follow up real-time assessments directly with patients that indicate “poor” or “dissatisfied” ratings, to discover what happened. - Conduct patient focus groups. - Ask real-time questions via patient kiosks. - Use a “patient tracer” (i.e., a real or simulated patient) to evaluate the experience of making, scheduling, and undergoing an appointment. - Carry out spot surveys focused on key site access issues over several weeks. - Consider the access experiences and perceptions of patient family members. - Develop a local patient experience template to reflect the key patient experience goals, barriers, and facilitators at the site. |
| **References and further reading for the recommendation** | American Academy of Family Physicians, “Patient Satisfaction Survey,” webpage, 2015.  Health Quality Ontario, “Advanced Access and Efficiency Workbook for Primary Care,” Toronto, July 2011.  Institute for Healthcare Improvement, “Today’s Office Visit Survey Card,” webpage, undated-c.  Trustees of Dartmouth College, Improving Patient Access to Care, Hanover, N.H.: Dartmouth College, April 2, 2002. |
| **Priority Action #8** | **Maximization of the Ability to Manage Demand**  *Maximize primary care team members’ ability to proactively manage demand (e.g., alerts, reminders, and telephone contacts from patients on their panels) by optimizing provider visit schedules (e.g., through triage, prospective “scrubbing” of appointments to the extent appropriate given their training/licenses).* |
| **Recommendation 8.A**  *Initiation Level*: Can be initiated by midlevel managers, including unit managers, primary care site leaders, or care team leaders | **Train primary care site leaders and teams in four key principles of proactively managing demand, including:**   - analyzing site-level data on past demand to plan short-term supply (i.e., workforce) adjustments - enabling providers to achieve needed same-day continuity visit availability by right-sizing their panels and by using advanced access principles - integrating patient preferences into all aspects of access management - using data resources to track site-level and team-level access (e.g., through dashboards). |
| **Suggestions for implementing the recommendation** | **Suggestions for training of primary care site leaders and teams include developing and implementing:**   - an open, engaged, problem-solving culture, not a punitive culture, around improving access - flexibility relative to local primary care site limitations and resources and on how to get assistance for problems implementing advanced access principles, especially when overall local primary care site workforce supply is inadequate to meet demand - use of advanced access principles, including adjusting return visit rates, enabling same-day continuity access, and matching provider expected and actual availability to see patients - support for use of non-face-to-face encounter methodologies (see also recommendation #3.A,B, on telephone access and #7.A on patient experience) - procedures for addressing patient preferences in arranging either face-to-face or non-face-to-face visits, especially when trade-offs between appointment promptness and desire to see a continuity team member are required - scripts to let the patient decide whether to wait for his or her provider to return from a scheduled leave or to be scheduled with his or her team or a different provider - patient education about the team approach and about alternative care options with team members - patient education about opportunities to contact the care team (e.g., telephone, email, or a patient portal) to enhance continuity (see Recommendation #3b,C on Telephone Access and #7A on Patient Experience).   **Suggestions for anticipating demand and adjusting supply include the following:**   - Measure past demand and use data to predict demand daily, weekly, and seasonally and to schedule supply (i.e., primary care workforce) accordingly. - Distinguish between activity (number of patients seen) and demand (number of requests for care), and focus on meeting the demand rather than the amount of activity. - Monitor overflow visits during periods of low access, such as preventable emergency department or urgent care visits, and account for the demand these patients represent. - Identify opportunities and plan for group visits or consultations with patients requesting or needing frequent visits (e.g., patients with chronic illnesses). - Develop roles for team members other than providers for subpopulations of patients (e.g., those with congestive heart failure, those with hyperlipidemia, or those using anticoagulation). - Plan for nurse practitioners and physician assistants to see patients of absent providers and/or to have their own panels. - Plan to spread high demand times, such as for school physicals and flu shots, over a longer period of time (e.g., schedule school physicals on the patient’s birthday). - Eliminate the distinction between routine and urgent appointments; distinguish, instead, between short and long (multiples of short) appointments (e.g., annual exams, new patients, chronic illness patients). - Measure nonappointment delays and efficiency (e.g., telephone message). - Plan to use tele-pharmacy, tele-primary care, and tele–mental health hubs. - Encourage providers to accomplish as much as possible during patient visits, anticipate patient near-term visit needs, and select follow-up visit intervals based on patient needs. - Assess management of telephone care (see Recommendations #3.A,B,C on telephone access)**.** |
| **References and further reading for the recommendation** | Health Quality Ontario, “Predict the Expected: Contingency Plans to Manage Advanced Access Schedules,” Toronto, undated.  Health Quality Ontario, “Advanced Access and Efficiency Workbook for Primary Care,” Toronto, July 2011.  Mehrotra, Ateev, Lori Keehl-Markowitz, and John Z. Ayanian, “Implementation of Open Access Scheduling in Primary Care: A Cautionary Tale,” Annals of Internal Medicine, Vol. 148, No. 12, 2008, pp. 915–922.  Murray M, Berwick DM. Advanced access: reducing waiting and delays in primary care. JAMA. 2003;289(8):1035-1040. |

*The recommendations and suggestions in this table are based on Stakeholder Panel discussion, ratings and consensus, informed by systematic review of access management intervention literature (see Miake-Lye IM, Mak S, Shanman R, Beroes JM, Shekelle PG. Access Management Improvement: A Systematic Review. *VA Evidence-based Synthesis Program Reports.* 2017; VA ESP Project #05-226, see https://www.hsrd.research.va.gov/publications/esp/AccessMgt.pdf) and by analysis of qualitative data on group practice management (see Hempel S, Stockdale S, Danz M, et al. *Access Management in Primary Care: Perspectives from an Expert Panel.* Santa Monica, CA: RAND Corporation 2018, available at <https://www.rand.org/pubs/research_reports/RR2536.html> ). The references in this table result from searching panel transcripts and final articles from the systematic review, with a few additional articles from informal literature search. A full systematic review covering each of the topics the panel considered important was beyond the project’s scope.
