# Supplementary material for: Eight Priorities for Improving Primary Care Access Management in Healthcare Organizations: Results of a Modified Delphi Stakeholder Panel
Source: J Gen Intern Med. 2019 Nov 14;35(2):523–30. doi: 10.1007/s11606-019-05541-2 (PMC7018673; doi:10.1007/s11606-019-05541-2)
Supplement: Supplementary file 2 — (DOCX 6279 kb) [file 11606_2019_5541_MOESM2_ESM.docx]

SLIDEDOC

EIGHT PRIORITIES FOR IMPROVING ACCESS MANAGEMENT

A Tool for **Supporting Learning Health Systems**

# About This Access Management Improvement Tool

*Eight Priorities for Improving Access Management*

##### OVERVIEW OF CONTENT

**PICK A PRIORITY**

**IDENTIFY RELEVANT**

**RECOMMEDNATIONS FOR ACTION**

Review Associated Suggestions and References

**#1**

- Suggestions
- Relevant

literature

**CHOSEN**

**PRIORITY**

**#2**

- Suggestions
- Relevant literature

**#3**

- Suggestions
- Relevant literature

[**SLIDES:**](#_bookmark3)

[**10-17**](#_bookmark3)

[**SLIDES:**](#_bookmark3)

[**10-17**](#_bookmark3)

[**SLIDES:**](#_bookmark2)

[**08-09**](#_bookmark2)

Access Management Definitions

2 Organizational Structure Targets

4 process Improvement Targets

2 Outcome Targets

**EIGHT PRIORITIES FOR**

**ACCESS IMPROVEMENT**

[**SLIDES:**](#_bookmark1)

[**03-07**](#_bookmark1)

Purpose of the tool

Why consult this tool

Methods used to develop the tool

Using the tool to develop an access management improvement agenda

Methods for working with stakeholders

**INTRODUCTION**

**I N T R O D U C T I O N**

**Purpose of the Tool**

**Purpose**:

This tool is intended to support access management improvement in primary care by providing evidence-based priorities and recommendations for achieving them to health care organization.

- The tool can be used to develop an organization- or primary care site- specific implementation agenda for access management improvement

**What the Tool Provides:**

For each of eight access management priorities identified through a modified Delphi stakeholder panel, the tool identifies recommendations for action, concrete suggestions for implementation, and references from the literature.

- The tool specifies the organizational level at which recommended changes are best

initiated (executive vs mid-level or frontline).

## How to Use the Slidedoc* Tool:

SlideDocs are meant to be read, rather than presented. Each slide summarizes key points and includes links to more detailed information through embedded links. For example, a link on [slide 9](#_bookmark2) provides users with a modifiable slide presentation on the eight priorities.

*Fisher CD. Slidedocs: Spread Ideas With Effective Visual Documents, by Nancy Duarte. *Academy of Management Learning & Education* 2017;16(2):339-42. doi: 10.5465/amle.2017.0112

*Eight Priorities for Improving Access Management*

**I N T R O D U C T I O N**

# Why consult this tool?

###### Effective access to primary care is critical for ensuring population health. Yet little research information addresses the ongoing challenges of access management.

*Eight Priorities for Improving Access Management*

Like many other policy issues, access management can be considered a “wicked” problem that is not universally addressable by applying any single formula or mandate. In part, this is because the achievement of optimal access must reflect a balancing of multiple local or organization-specific factors. In addition, access management improvement must be ongoing, because local circumstances continuously change, including fundamental aspects of access such as the size of the population an organization aims to serve.

This tool assumes that 1) evidence from scientific literature and from the experiences of high quality healthcare organizations can be helpful for improving access management; 2) identifying key priorities for organizations to consider can enable better improvement agendas; and 3) input from an organization’s stakeholders will be necessary to shape the actions taken for achieving each chosen priority.

**I N T R O D U C T I O N**

**Methods Use to Develop the Tool**

**We assembled evidence on group practice management by:**

- Carrying out systematic evidence review, reviewing access manuals, and conducting qualitative study of group practice managers

**We carried out a modified Delphi stakeholder panel by:**

- Recruiting, based on an established panelist identification frame, 20 diverse stakeholders with potentially competing interests. Panelists included health care consumers, providers, payers, purchasers, researchers, product makers, and policymakers from the US
- Using written pre- and post- panel surveys, anonymous electronic voting, and meeting transcripts to collate individual stakeholder input, revise preliminary documents, and to assess the level of consensus independent from potential group pressure
- Engaging panelists in defining access management and using qualitative content analyses, including word clouds based on panel transcripts, to identify themes and revise final definitions

## All panelists reviewed final materials for approval.


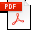


[RAND Report](https://www.rand.org/pubs/research_reports/RR2536.html)

*Eight Priorities for Improving Access Management*

**I N T R O D U C T I O N**

# Using the Tool to Develop an Access Management Improvement Agenda

###### Working with stakeholders to develop an access management improvement agenda can improve the likelihood that robust ongoing achievement of access goals will be achieved. The agenda should span organizational boundaries and take account of local progress and resources. Agenda development based on this tool may include:

- Identification of key stakeholders and their roles in access management improvement
- Self-Assessment of the organization or site’s progress relative to the eight

priorities

- Development of an organization-specific flow chart and logic model

*(see embedded examples)*

- Choosing priorities and the linked recommendations for action *(see embedded*

*slides on assessing and implementing an access improvement agenda)*

- Using the suggestions and references for each chosen recommendation to inform

a specific quality improvement plan’s aims and measures

**Access Flow Chart Access Logic Model Assessing and Implementing**

**Access Agenda**

*Eight Priorities for Improving Access Management*

**I N T R O D U C T I O N**

# Methods for Working with Stakeholders

## Below is an example approach

*Eight Priorities for Improving Access Management*

1. Identify key stakeholder groups: e.g., by type (health care consumers, providers, payers, purchasers, researchers, product makers, and policymakers); by expertise (e.g., call centers); by organizational level (e.g., executive, middle manager, front line) and by professional or academic discipline (e.g., nurse, physician, administrator, subject expert). Consider how you want to engage these (e.g., as part of a broader advisory group; as part of a small Quality Improvement or QI team)
2. Have stakeholders assess each of the 8 priorities in terms of the degree to which the organization as achieved the priority (supply local data if possible)
3. Pick a priority to start with, and engage a QI team in reviewing the recommendations and suggestions associated with the chosen priority in the tool
4. Have the QI team use these to develop aims, measures, methods and timeline for

achieving these, and budget including release time

# Additional Literature

#### The RAND Research Report is now available online, including a full set of

**references**

<https://www.rand.org/pubs/research_reports/RR2536.html>

#### Additional examples from the literature relevant to implementing an access

**management improvement implementation agenda include:**

- - **Importance of agenda-setting:** Clack L, Zingg W, Saint S, et al. Implementing infection prevention practices across European hospitals: an in-depth qualitative assessment. *BMJ quality & safety* 2018;27(10):771-80.
  - **How to identify representative stakeholders** Concannon TW, Meissner P, Grunbaum JA, et al. A new taxonomy for stakeholder engagement in patient-centered outcomes research. *J Gen Intern Med* 2012;27(8):985-91. doi: 10.1007/s11606-012-2037-1
  - **How to carry out a priority-setting panel Rubenstein LV**, Fink AR, Yano EM, et al. Increasing the impact of quality improvement on health: an expert panel method for setting institutional priorities. Joint Commission Journal on Quality Improvement 1995; 21(8):420-432
  - **Developing QI Team Aims and Measures:** Quality Improvement Essentials Toolkit, Institute for Healthcare Improvement, Boston, Massachusetts, USA<http://www.ihi.org/Topics/ImprovementCapability/Pages/default.aspx>
  - **Responsive evidence review for quality improvement innovations:** Danz MS, Hempel S, Lim Y, et al. Incorporating evidence review into quality improvement: meeting the needs of innovators. BMJ Qual Saf. 2013 Nov;22(11) Epub 2013 Jul 5 PMID: 238322925

*Eight Priorities for Improving Access Management*

**Eight Priorities for Access Management**

**Organizational Structure Targets**

- Interdisciplinary primary care site leadership
- Clearly identified group practice management structure

**Process Improvement Targets**

- Patient telephone access management
- Contingency staffing
- Nurse management of demand through care coordination
- Optimizing provider visit schedules

**Outcome Targets**

- Quality of patients’ experiences of access
- Provider and staff morale in relationship to supply-demand mismatch

**Presentation: Access Management**

**E I G H T P R I O R I T I E S**

**Priorities in Primary Care**

*Eight Priorities for Improving Access Management*

**Access Management Quality Improvement Tool from**

**https://**[**www.rand.org/pubs/research_reports/RR2536.html**](http://www.rand.org/pubs/research_reports/RR2536.html)

**O R G A N I Z A T I O N A L S T R U C T U R E T A R G E T**

# Interdisciplinary Primary Care Site Leadership

### Identify physician, registered nurse, and administrative leaders for each primary care practice site with authority to support access management priorities within local site contexts.

*Eight Priorities for Improving Access Management*


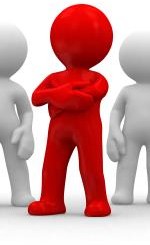


**O R G A N I Z A T I O N A L S T R U C T U R E T A R G E T**

# Group Practice Management Structure

### Develop a clearly identified group practice management structure with a designated group practice manager who reports to executive leadership, communicates with individual primary care sites, and can collaborate across roles and service lines (e.g., medicine, nursing, administration).

*Eight Priorities for Improving Access Management*


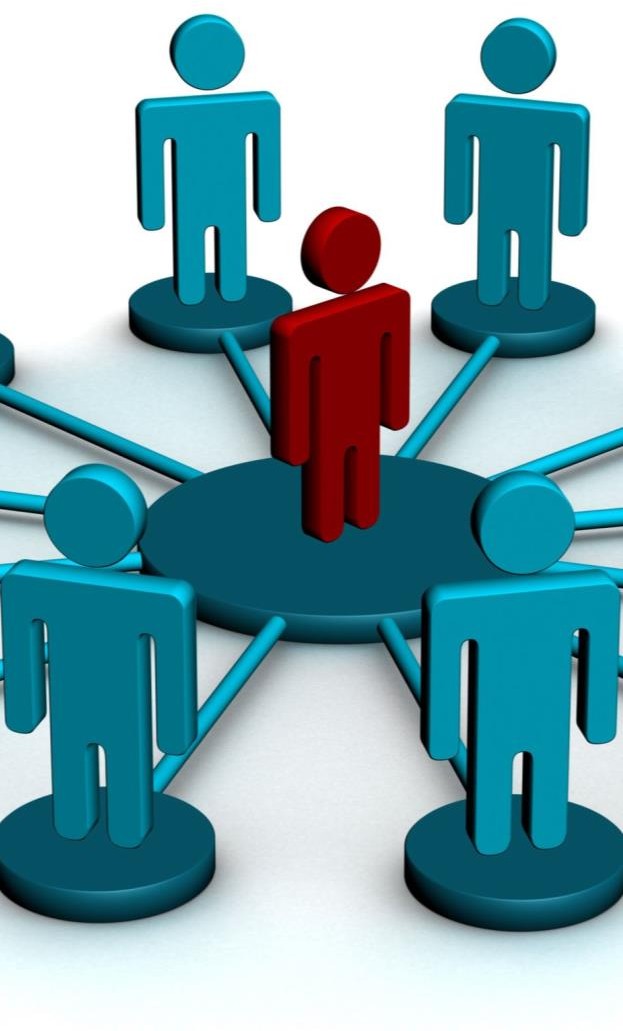


**P R O C E S S I M P R O V E M E N T T A R G E T**

# Patient Telephone Access Management

### Routinely evaluate the degree to which patient telephone calls are (a) answered promptly and

*Eight Priorities for Improving Access Management*


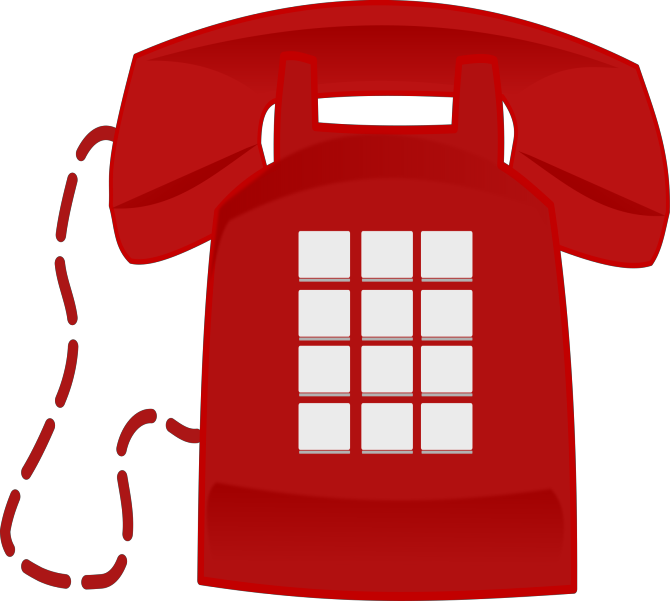
(b) routed accurately and appropriately, as judged in

terms of patients’ clinical needs

and preferences

**P R O C E S S I M P R O V E M E N T T A R G E T**


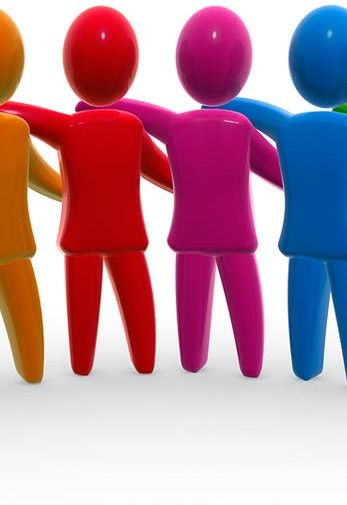
**Contingency Staffing**

### Maximize access managers’ routine use or ability to demonstrate systematic approaches to ensuring adequate availability of contingency staffing (i.e., planned minimal excess staffing to cover routine absences, such as those resulting from hiring gaps, vacations, illness).

*Eight Priorities for Improving Access Management*

**P R O C E S S I M P R O V E M E N T T A R G E T**

**Nurse Management of Demand Through Care Coordination**

Maximize the ability of the primary care team’s registered nurses to prospectively manage demand by leading care coordination for their panels.

*Eight Priorities for Improving Access Management*


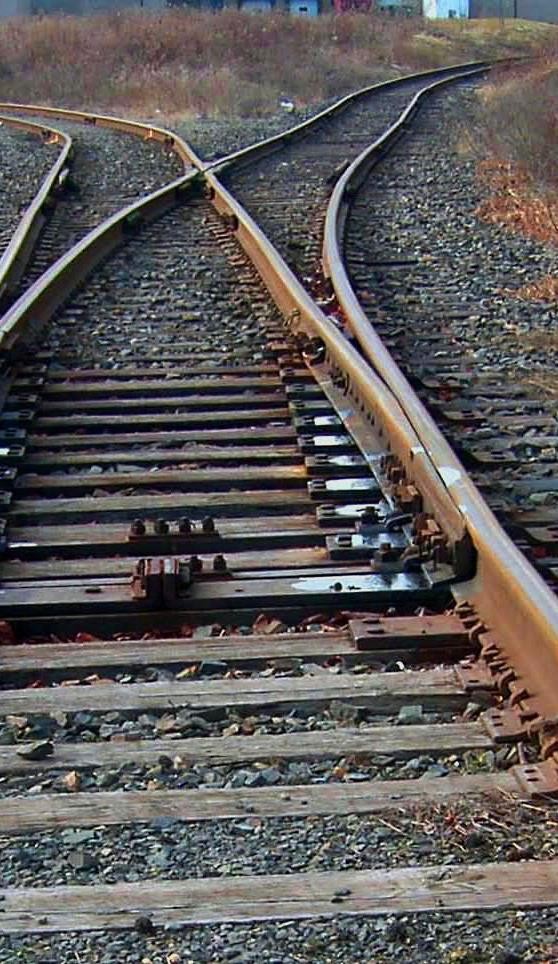


**P R O C E S S I M P R O V E M E N T T A R G E T**

# Proactive Demand Management By Optimizing Provider Visit Schedules

### Maximize primary care team members’ ability to proactively manage demand (e.g., alerts,

*Eight Priorities for Improving Access Management*


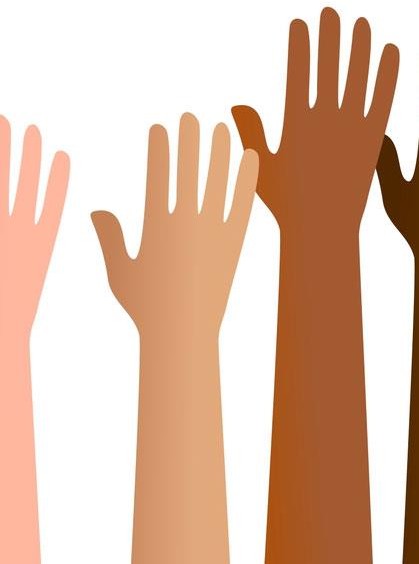
reminders, and telephone contacts from patients on their panels) by optimizing provider visit schedules (e.g., through triage, prospective “scrubbing” of appointments to the extent appropriate given their training/licenses).

**O U T C O M E S T A R G E T**


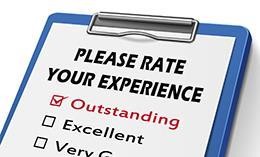
**Quality of Patients’ Experiences of Access**

### Assess the quality of the patient’s experiences of access (i.e., patient- rated access). We expect patient ratings to reflect both in-person and non-face-to-face (e.g., telehealth, telephone, secure messaging) care.

*Eight Priorities for Improving Access Management*

**O U T C O M E S T A R G E T**


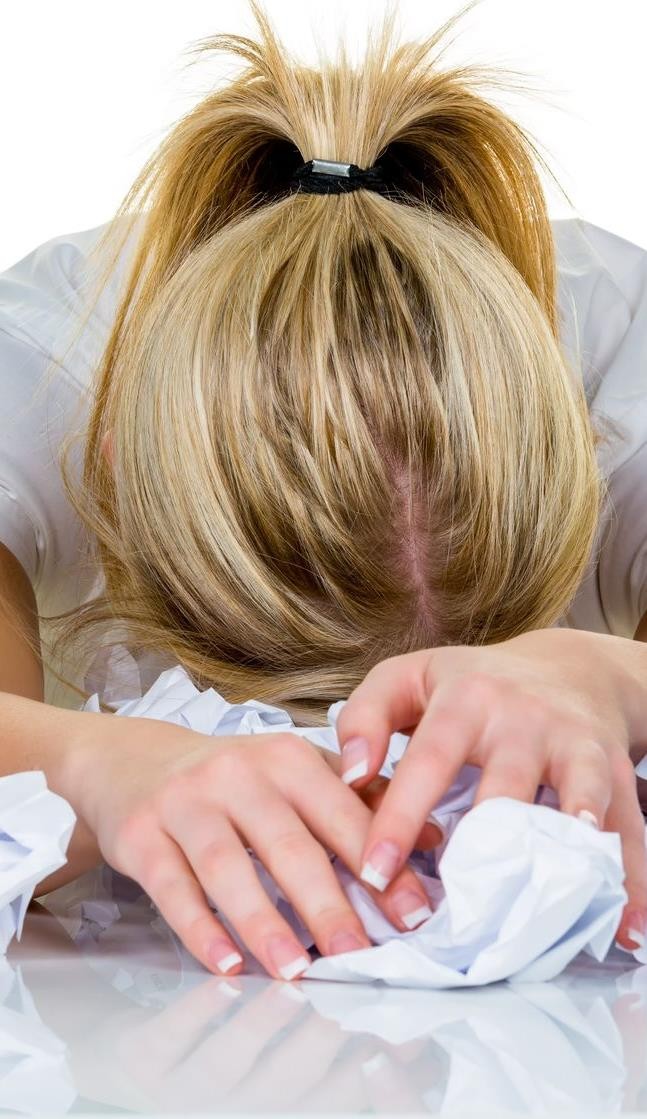
**Provider and Staff Morale in Relationship to Supply-Demand Mismatch**

### Assess primary care provider and staff morale (e.g., low/high burnout, job satisfaction, or turnover rates) in relation to supply-demand mismatch (e.g., panels exceeding recommended size, primary care provider vacancies).

*Eight Priorities for Improving Access Management*
